# Multimodal investigation of dacomitinib–calf thymus DNA binding interaction: insights from spectroscopy, thermodynamics, and *in-silico* studies

**DOI:** 10.1039/d5ra09242f

**Published:** 2026-01-26

**Authors:** Manal A. Alossaimi, Taibah Aldakhil, Heba Elmansi, Fathalla Belal, Galal Magdy

**Affiliations:** a Pharmaceutical Chemistry Department, College of Pharmacy, Prince Sattam bin Abdulaziz University Al-Kharj 11942 Saudi Arabia m.alossaimi@psau.edu.sa; b Pharmaceutical Analytical Chemistry Department, Faculty of Pharmacy, Mansoura University Mansoura 35516 Egypt; c Pharmaceutical Analytical Chemistry Department, Faculty of Pharmacy, Kafrelsheikh University Kafrelsheikh 33511 Egypt galal_magdy@pharm.kfs.edu.eg; d Department of Pharmaceutical Analytical Chemistry, Faculty of Pharmacy, Mansoura National University Gamasa 7731168 Egypt

## Abstract

Dacomitinib is a second-generation tyrosine kinase inhibitor (TKI) used as a first-line targeted therapy for patients with metastatic non-small cell lung cancer. In this study, its interaction with calf thymus DNA (ctDNA) was systematically investigated using UV-Vis spectrophotometry, spectrofluorimetry, viscosity measurements, ionic strength variation, thermodynamic analysis, molecular docking, and molecular dynamics (MD) simulations. The results revealed a strong binding affinity between dacomitinib and ctDNA, with a preferential minor groove-binding mode confirmed through competitive displacement assays using ethidium bromide and rhodamine B, and further supported by UV-Vis and viscosity data. The binding constant (*K*_b_) at 298 K, calculated using the Benesi–Hildebrand equation, was 7.7 × 10^5^ M^−1^, indicating high affinity. Thermodynamic parameters (Δ*H*° and Δ*S*°) indicated that the interaction is primarily governed by van der Waals forces and hydrogen bonding. Frontier molecular orbital (HOMO/LUMO) analysis of dacomitinib showed a wide energy gap, reflecting a favorable tendency for biomolecular interaction. Molecular electrostatic potential (MEP) mapping identified electrophilic and nucleophilic regions and predicted preferential non-covalent interaction sites. Non-Covalent Interaction (NCI) analysis revealed a compact network of van der Waals contacts with minor steric repulsion, supporting conformational stability. Molecular docking confirmed strong, directional, non-covalent affinity toward the DNA minor groove. MD simulations over 100 ns demonstrated structural stability of the dacomitinib–ctDNA complex through stable RMSD, reduced RMSF, compact radius of gyration, and persistent hydrogen bonding. This study provides an integrated experimental and computational analysis of the interaction between dacomitinib and ctDNA, elucidating a non-intercalative minor groove binding mode and offering mechanistic insight into how a targeted kinase inhibitor can engage DNA through shape complementarity, hydrogen bonding, and van der Waals interactions. The observed interaction reflects a binding interaction under *in vitro* conditions and is not intended to imply a direct role in the established anticancer mechanism of dacomitinib. Beyond characterizing a specific drug–DNA system, these findings highlight general principles governing minor groove recognition by non-classical DNA-binding small molecules and underscore the importance of evaluating potential off-target nucleic acid interactions of targeted therapeutics.

## Abstract 2

An integrated experimental and computational study reveals that dacomitinib preferentially binds calf thymus DNA *via* minor groove interactions, providing new insight into non-covalent DNA engagement by kinase inhibitors.

## Graphical abstract

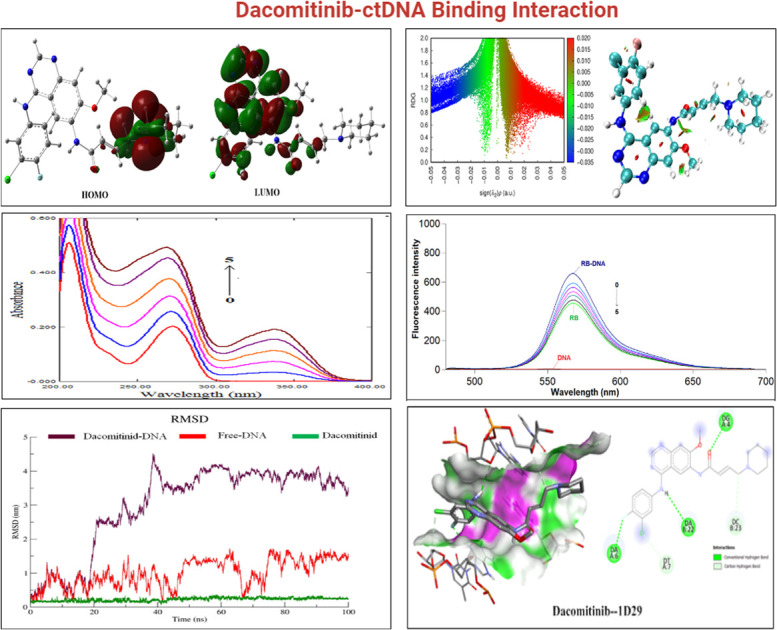

## 1. Introduction

In recent decades, the high incidence and mortality rates of cancer have driven significant advancements in the development of cytostatic and cytotoxic chemotherapeutic drugs. Certain anti-tumor agents exert their effects by binding to DNA in cancer cells, leading to either suppressed cell growth (cytostatic or antiproliferative action) or cell death (cytotoxic action). The therapeutic effectiveness of these drugs is closely tied to their binding mechanisms and the nanoscale interactions with DNA. Consequently, a detailed understanding of these biophysical properties particularly in terms of molecular recognition plays a crucial role in optimizing drug design and improving medical treatment strategies.

**Fig. 1:**
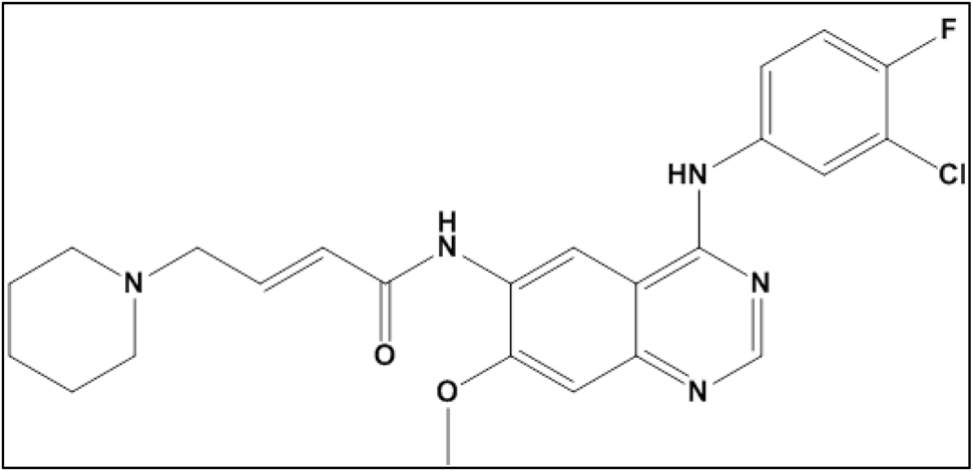
Chemical structure of dacomitinib.

Dacomitinib (DCN), chemically designated as (2*E*)-*N*-[4-[(3-chloro-4-fluorophenyl)amino]-7-methoxyquinazolin-6-yl]-4-(piperidin-1-yl)but-2-enamide, is an orally administered, highly selective second-generation tyrosine kinase inhibitor (TKI) (Fig. 1). As an irreversible inhibitor, it binds covalently to the ATP-binding site of epidermal growth factor receptor (EGFR) family kinases, distinguishing it from reversible first-generation TKIs.^1^ Pfizer Inc. developed DCN, which the FDA approved on September 27, 2018. Although more research is required, some evidence in the literature points to DCN's potential as a treatment in the epithelial ovarian cancer model.^2^

Investigating the binding interactions between DNA and pharmaceutical compounds such as DCN is essential for deciphering the molecular mechanisms underlying drug effectiveness and potential cytotoxic effects. Such studies provide critical insights into how therapeutic agents engage with genetic material, which may modulate vital cellular processes including transcription regulation, DNA replication fidelity, and damage repair pathways. To characterize these interactions at a molecular level, a multifaceted experimental approach has been employed, incorporating: spectroscopic analyses to detect conformational changes and binding constants, hydrodynamic measurements as viscosity to assess DNA structural perturbations, thermodynamic profiling to determine the energetics (Δ*G*, Δ*H*°, Δ*S*°) and driving forces of association, electrostatic evaluations through ionic strength variation experiments and computational modeling through molecular docking and molecular dynamics to predict binding motifs, affinity, and stability. This integrative methodology enables comprehensive elucidation of drug–DNA binding modalities, ranging from intercalation and groove-binding to covalent adduct formation. The resulting data not only clarify structure–activity relationships but also inform pharmacological optimization by recognizing potential genotoxic risks or therapeutic synergies.^3–6^

Deoxyribonucleic acid (DNA) serves as the fundamental molecular blueprint of life, orchestrating vital biological processes through precise control of genetic transcription and replication. These mechanisms govern the biosynthesis of functional proteins and enzymes essential for cellular homeostasis. The structural and dynamic interactions between DNA and small ligand molecules constitute a critical area of pharmaceutical research, providing a molecular framework for rational drug design. Such investigations facilitate the development of targeted therapeutics with optimized binding affinity, pharmacological efficacy, and biological specificity, while minimizing off-target effects.^7–9^

DNA-binding studies commonly employ several model systems, including calf thymus DNA (ctDNA),^7–12^ salmon sperm DNA,^13–15^ and herring sperm DNA.^16,17^ These models exhibit similar structural properties, particularly in their base pair length (typically 587–831 bp) and nucleotide distribution, containing about 41.9% G-C and 58.1% A-T base pairs. Given these comparable features, all three DNA types demonstrate nearly identical physicochemical behavior.^18^ Among these, ctDNA has emerged as the most prevalent and standardized model system for DNA interaction studies.

DNA–ligand interactions occur through both covalent and non-covalent binding mechanisms, with non-covalent associations representing the predominant mode of molecular recognition. These interactions can target distinct structural domains of the DNA double helix, including: the narrow minor groove, the wider major groove, intercalative insertion between stacked base pairs, external surface binding along the phosphodiester backbone, and long-range electrostatic attractions with charged groups. Each binding mode exhibits characteristic molecular recognition patterns and confers unique effects on DNA structure and function.^19^ Different studies have been reported to inspect the binding mode of drugs with DNA.^4,11,20^ This type of research helps us in the development of new drugs, disease diagnosis, and creating new biotechnologies.

Recent studies have investigated dacomitinib's binding characteristics with proteins such as human α1-acid glycoprotein using multispectral and computational modeling, revealing that its interactions are mainly stabilized by hydrogen bonding and hydrophobic forces.^21^ However, these findings pertain to protein targets, not DNA.

With the aid of computational tools, the discovery of specific anticancer agents continues to progress, by deepening the understanding into molecular interaction mechanisms even before new compounds are tested *in vitro*. Modeling approaches, such as quantum chemistry, molecular docking and molecular dynamics, are now indispensable elements in the rational design of drugs.^22,23^ These methods permit the electronic properties, chemical reactivity, as well as the binding patterns of ligands with the biomolecules to be characterized, in particular with DNA. In this regard, second-generation inhibitors of EGFR have been developed, and among them DCN (anilinoquinazoline) has entered clinic trials. It exerts an irreversible action by forming a covalent bond with the Cys797 residue of the tyrosine kinase domain, and inhibits the pathways responsible for cell proliferation, differentiation, and tumor survival. In contrast to other reversible inhibitors such as gefitinib or erlotinib, DCN has a pan-HER inhibition profile by simultaneously inhibiting EGFR, HER2 and HER4, resulting in its better clinical efficacy in non-small cell lung cancers (NSCLC) with activating EGFR mutations.^24^ However, in addition to its classical enzymatic pathway, it is manifest, from recent evidences, that DCN might also interact with non-canonical targets, namely DNA, where it binds non-covalently to the minor grooves. Such a hypothesis is supported by *in silico* studies that show the compound's propensity to insert into the ctDNA double helix and form weak interactions (van der Waals and hydrogen bonds) of potential interest in gene expression or structural stability of the ctDNA.

In this context targeting with more temporal or depth provides a need for a multimodality profiling of DCN. Frontier molecular orbital (HOMO/LUMO) analysis can be employed to ascertain the potential reactive site of drug molecule,^25^ and molecular electrostatic potential (MEP) mapping shows the site of high charge density which may be convenient to interact with the nucleic acid bases through electrostatic interaction.^26^ Moreover, the Non-Covalent Interactions (NCI) method^27^ offers opportunities to visualize weak forces determining conformational stability in an intricate biological milieu. These calculations are compared with molecular docking simulations for the determination of the binding free energy of DCN into different DNA forms and molecular dynamics calculations over tens of nanoseconds for the evaluation of the stability of the complex in a condition simulating the intracellular environment.^28^

In the present study, therefore, an attempt has been made to reveal the dual profile of a synthesized ligand such as DCN (which has clearly noticeable activities against different types of cancers): (I) at the level of cell based bioelectronics interactomics, and (II) by adopting more than one mode of action which will eventually target the membrane-bound HER receptors, and nuclear DNA systems through combinatorial approach based on the physicochemical properties of the ligand. A similar, combined clinical and molecular simulations approach could drive new personalized therapies with increased selectivity and reduced systemic side effects.

Drug–DNA interactions are frequently too complex to fully grasp with a single approach. In order to facilitate the processes of drug development and regulatory approval, it is essential to create quick, high-throughput, continuous, and economical methods for assessing how different medicines interact with DNA. The current work aimed to thoroughly examine the relationship between DCN and ctDNA utilizing a variety of methodologies. Viscosity, ionic strength tests, UV-visible spectroscopy, fluorescence spectroscopy, and thermodynamics are some of these methods. Molecular docking and molecular dynamics simulations were also performed to examine the specifics, such as the binding mode, binding forces, and interaction locations. Although multispectroscopic techniques combined with molecular docking and molecular dynamics simulations are now routinely employed to study small molecule, DNA interactions, their application remains valuable when used to address mechanistic questions extending beyond simple binding confirmation. In this context, DCN represents a chemically and pharmacologically relevant system, as it is an irreversible EGFR tyrosine kinase inhibitor designed for selective protein targeting rather than nucleic acid recognition. Investigating its interaction with DNA therefore provides an opportunity to examine how molecular shape, rigidity, and functional group distribution, features optimized for protein binding, can nevertheless promote non-covalent association with the DNA minor groove. Such analyses are particularly relevant for understanding off-target interactions of modern targeted therapeutics and for distinguishing groove binding from intercalative or electrostatically driven binding modes. Accordingly, the present work aims not only to characterize the DCN–DNA interaction but also to contribute to broader chemical and biophysical principles governing DNA recognition by non-classical small molecules.

## 2. Experimental

### 2.1. Materials and chemicals

Dacomitinib (% purity 99.89) was obtained from the European division of Pfizer (Europe MA EEIG).

Sigma Aldrich (St. Louis, MO, USA) provided a variety of chemicals, such as ctDNA, Tris–HCl, Rhodamine B (RB), and ethidium bromide (EB).

A solution of 0.05 M Tris–HCl buffer (pH = 7.4) was made using distilled water.

Tris–HCl buffer was used to obtain the ctDNA stock solution. There was no protein contamination in ctDNA, as evidenced by the DNA's *A*_260_/*A*_280_ ratio of 1.99 (above 1.8).^11,29^ The absorbance value at 260 nm (*ε*_260_ = 6600 M^−1^ cm^−1^, *T* = 298 K) was used to calculate the concentration of the ctDNA stock solution. The produced DNA solutions were used within five days and stored at 4 °C ethidium bromide (EB) (1.2 × 10^−3^ M) and rhodamine B (RB) (2.0 × 10^−3^ M) were made by dissolving them in ethanol and keeping them at 4 °C.

### 2.2. Instrumentation

The experimental investigation employed the following analytical instrumentation:

UV-Vis Spectrophotometry: absorption measurements were conducted using a T80+ UV/VIS PC spectrophotometer (PG Instruments Ltd, UK) equipped with 1.0 cm path length quartz cuvettes for optimal spectral resolution.

Fluorescence Spectroscopy: emission measurements were performed on a Cary Eclipse spectrofluorimeter (Agilent Technologies, USA) featuring a pulsed Xenon light source for enhanced sensitivity and reduced photobleaching effects.

Viscometric Analysis: solution viscosity measurements were acquired using an Oswald capillary viscometer maintained at 298.0 ± 0.1 K, with precise flow kinetics monitored through a 0.57 mm internal diameter capillary tube.

### 2.3. Investigation of DNA binding interaction

#### 2.3.1. Spectrophotometric approach

A fixed concentration of ctDNA (3.0 × 10^−5^ M) was titrated with incremental additions from DCN (0–10.5 × 10^−6^ M). Spectral scans (200–400 nm) were recorded at three controlled temperatures: 298 K (25 °C), 303 K (30 °C), and 313 K (40 °C). Binding constants (*K*_b_) were calculated *via* Benesi–Hildebrand plots. For ionic strength modulation; fixed concentrations of DCN (6.3 × 10^−6^ M) and ctDNA (3.0 × 10^−5^ M) were prepared. NaCl concentration systematically varied (0–0.07 M) in 0.01 M increments. Absorbance measurements were taken at 260 nm for DCN–ctDNA complex. The average of three measurements of each spectrum was obtained (*n* = 3).

#### 2.3.2. Spectrofluorimetric approach

Fluorescence probes including RB and EB were examined at concentrations and wavelengths; 2.0 × 10^−3^ M, *λ*_ex_/*λ*_em_ = 465/577 nm and 1.2 × 10^−3^ M, *λ*_ex_/*λ*_em_ = 525/624 nm, respectively. The emission spectra were scanned after mixing ctDNA (15.0 µM) with each of them, in the presence and absence of DCN increasing concentrations (0–10.5 × 10^−6^ M). The average of three measurements of each spectrum was obtained (*n* = 3).

#### 2.3.3. Viscosity assessments

The experiment was conducted at 298 K by adding different DCN concentrations (0–10.5 µM) to 30.0 µM ctDNA in Tris–HCl buffer. A stopwatch was used to record the flow time by taking an average of three determinations (*n* = 3). Using the formula (*η* = (*t* − *t*_0_)/*t*_0_), where (*t*) is the measured flow time of ctDNA-containing solutions and (*t*_0_) is the flow time of Tris–HCl buffer alone, the viscosity was computed. Relative specific viscosity (*η*/*η*_0_)^1/3^ was then determined, where *η* and *η*_0_ denote the ctDNA specific viscosities with and without DCN, respectively.^11,30^. (*η*/*η*_0_)^1/3^*versus* binding ratio *r* (*r* = [DCN]/[ctDNA]) was used to graph the obtained results.

#### 2.3.4. Computational techniques methodology

Dacomitinib electronic and interactional properties were analyzed by means of multilevel *in silico* analysis. The geometry of DCN was initially optimized using density functional theory (DFT) at the B3LYP level of theory with 6-31G(d,p) basis set using Gaussian 09 package to achieve a stable minimum energy conformation.^31^ According to these optimized structure, we have calculated the frontier molecular orbitals (HOMO and LUMO), energy gap and molecular electrostatic potential (MEP) for evaluating chemical reactivity and to predict possible sites of electrophilic and nucleophilic interactions.^25,26^

The Non-Covalent Interaction (NCI) analysis has been done by the method of Reduced Density Gradient (RDG) described by Johnson *et al.*^27^ by taking advantage of Multiwfn and VMD software. This presentation combined makes the weak interactions (van der Waals forces, hydrogen bonds and steric repulsions) visible as colored isosurface, which gives a qualitative impression on the conformational stability of the ligand.

To examine the capacity of DCN to directly interact with DNA, molecular docking studies of dacomitinib's binding to canonical double-stranded DNA were performed, using AutoDock Vina^27^ and known DNA structures (PDB IDs: 1BNA, 1D29, 3EY0 and 6ASF). The ligand was generated with Open Babel and Gasteiger partial charges were assigned and rotatable bonds were defined. Docking box was for the DNA minor groove and generated poses were ordered based on binding energy and the structural similarity of the interactions (hydrogen bond, hydrophobic contact, π–π stacking) found. Finally, the dynamic stability of DCN–DNA complex was studied by 100 ns molecular dynamic (MD) simulation employing GROMACS software.^32^ The system was added the water (SPC/E), counterions for its charge neutrality and the simulation was performed under NPT conditions (temperature: 300 K, pressure: 1 atm) with the force field of AMBER99SB.^33–35^ Post simulation analysis centering on the root-mean-square deviation (RMSD), root-mean-square fluctuation (RMSF), radius of gyration (*R*_g_), solvent-accessible surface area (SASA), and number of hydrogen bond between ligand and DNA was used to evaluate the structural stability of complex during simulation. MD simulations were performed following standard equilibration protocols. Reported metrics, including RMSD, RMSF, *R*_g_, SASA, and hydrogen-bond profiles, were obtained from single, well-equilibrated production trajectories and are presented as representative exemplars. While each trajectory reflects a single run, the observed stability trends, such as RMSD convergence, preservation of DNA compactness, and persistent non-covalent interactions, are consistent with previously reported MD behavior for minor groove-bound DNA–ligand complexes. This limitation is noted to ensure transparency regarding reproducibility.

## 3. Results and discussion

Deciphering the molecular interactions between pharmaceutical compounds and DNA represents a fundamental research pursuit in drug discovery and biomedical sciences. As the repository of genetic information, DNA's association with therapeutic agents can critically modulate drug activity and biological effects. The present investigation systematically examines the binding dynamics of DCN with ctDNA through an integrative biophysical approach, incorporating advanced spectroscopic analyses, hydrodynamic assessments, thermodynamic profiling, and computational simulations. This comprehensive strategy enables precise characterization of the binding modality, association strength, and molecular recognition patterns, offering mechanistic insights that could inform rational drug optimization and enhance therapeutic potential.

### 3.1. Evaluation of binding mode of DCN with ctDNA

#### 3.1.1. Spectrophotometry

**Fig. 2:**
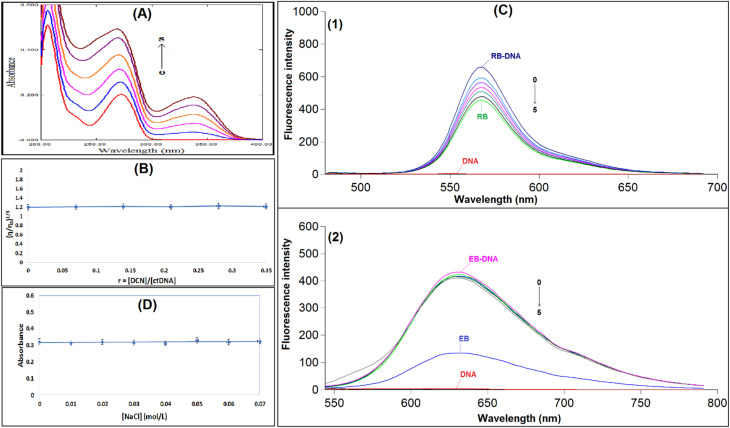
(A) UV absorption spectra of ctDNA (3.0 × 10^−5^ M) with increasing concentrations of DCN. The concentrations of DCN from 0 to 5 were 0, 2.1 × 10^−6^, 4.2 × 10^−6^, 6.3 × 10^−6^, 8.4 × 10^−6^, and 10.5 × 10^−6^ M, respectively. (B) Influence of variable concentrations of DCN (0–10.5 µM) on the viscosity of ctDNA (30.0 µM) in Tris–HCl buffer. (C) (1) Fluorescence emission spectra of the RB–ctDNA complex in presence and absence of DCN at 298 K. *C* (ctDNA): 30.0 µM; *C* (RB): 2.0 × 10^−3^ M; *C* (DCN) (0 → 5): 0, 2.1, 4.2, 6.3, 8.4, 10.5 µM, (*λ*_ex_/*λ*_em_ = 465/577 nm), (2) fluorescence emission spectra of the EB–ctDNA complex in presence and absence of DCN at 298 K. *C* (ctDNA): 30.0 µM; *C* (EB): 1.2 × 10^−3^ M; *C* (DCN) (0 → 5): 0, 2.1, 4.2, 6.3, 8.4, 10.5 µM, (*λ*_ex_/*λ*_em_ = 525/624 nm). (D) Influence of the ionic strength of NaCl on the absorbance of ctDNA–DCN complex. Concentrations of DCN and ctDNA were 6.3 × 10^−6^ M and 3.0 × 10^−5^ M, respectively. The concentrations of NaCl: 0, 0.01, 0.02, 0.03, 0.04, 0.05, 0.06, 0.07 M. Results are reported as mean ± standard deviation (SD) from three separate determinations (*n* = 3).

This approach records variations in the intensity and position of DNA's characteristic absorption peak at 260 nm, corresponding to the π–π* electronic transitions of nucleobases.^11,30^ As depicted in Fig. 2A and Table S1, incremental additions of DCN induced a progressive hyperchromic effect in ctDNA's absorption band without any spectral shift. The observed hyperchromic effect, coupled with the absence of peak displacement, suggests that DCN preferentially binds to ctDNA through minor groove interactions rather than intercalation.^30,36,37^

#### 3.1.2. Viscosity measurements

Conventional intercalative binding greatly alters the viscosity of DNA solutions because it necessitates significant separation between adjacent base pairs to extend the double helix and fit the drug.^38^ On the other hand, non-classical intercalation might cause the DNA helix to twist, shortening its length and decreasing viscosity. In comparison, electrostatic and groove binding interactions usually have little to no effect on DNA viscosity.^30,37–39^ The relative specific viscosity (*η*/*η*_0_)^1/3^ of ctDNA showed almost no change, as illustrated in Fig. 2B and Table S2, reinforcing the evidence that DCN preferentially interacts with ctDNA through groove binding. This result is consistent with the spectrophotometric data.

#### 3.1.3. Competitive binding studies

This method employs a well-characterized DNA-binding probe, such as ethidium bromide (EB) or rhodamine B (RB), whose interaction with DNA is well understood. These probes exhibit a strong increase in fluorescence upon binding to DNA.^16,40^ Studies have demonstrated that RB primarily binds to the minor groove of DNA, favoring AT-rich sequences, while EB intercalates directly between the base pairs.^41,42^

This study examined the interaction of DCN with DNA-bound EB and RB. When DCN was introduced to the DNA–EB complex, the fluorescence intensity remained largely unchanged, indicating no competitive binding between DCN and EB (Fig. 2C and Table S3). In contrast, increasing concentrations of DCN led to a gradual reduction in the fluorescence of the DNA–RB complex (Fig. 2C and Table S4). These findings suggest that DCN competes with RB for minor groove binding sites on ctDNA.

These results further confirm that DCN preferentially interacts with ctDNA by a minor groove binding mechanism rather than intercalation, aligning with the results obtained from spectroscopic and viscosity studies. Such assays are best interpreted as diagnostic tools for identifying dominant binding tendencies rather than definitive structural determinants.

#### 3.1.4. Ionic strength

The strength of electrostatic interactions is largely determined by the reaction medium's ionic strength. The interaction between ligands and macromolecules is facilitated by electrostatic forces, which are very mild under physiological conditions.^43^ The effect of NaCl concentration on the binding was investigated in order to evaluate the likelihood of electrostatic contact between ctDNA and DCN. When the NaCl concentration rose from 0 to 0.07 M, the absorbance of the ctDNA–DCN complex stayed almost constant, as seen in Fig. 2D and Table S5, indicating that there was no electrostatic interaction. Thus, there is favorably a minor groove binding interaction between DCN and ctDNA.

### 3.2. Evaluation of the binding affinities of ctDNA and DCN

Evaluating a drug's binding affinity to a biomacromolecule is crucial since there is an inherent relationship between the two. Either the dissociation constant (*K*_d_) or the binding constant (*K*_b_) can be used to measure the binding affinity. The *K*_b_ value for the 1 : 1 DCN–ctDNA complex can be found by applying the Benesi–Hildebrand eqn (1):^30,37^1
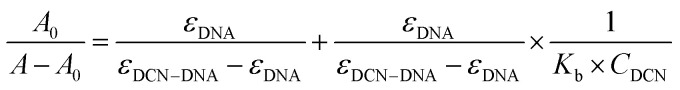
where: *A* and *A*_0_: ctDNA absorbance with and without DCN, respectively. *ε*_DNA_ and *ε*_DCN–DNA_: ctDNA and DCN–ct-DNA complex molar extinction coefficients, respectively. *C*_DCN_ is the DCN concentration.

**Fig. 3:**
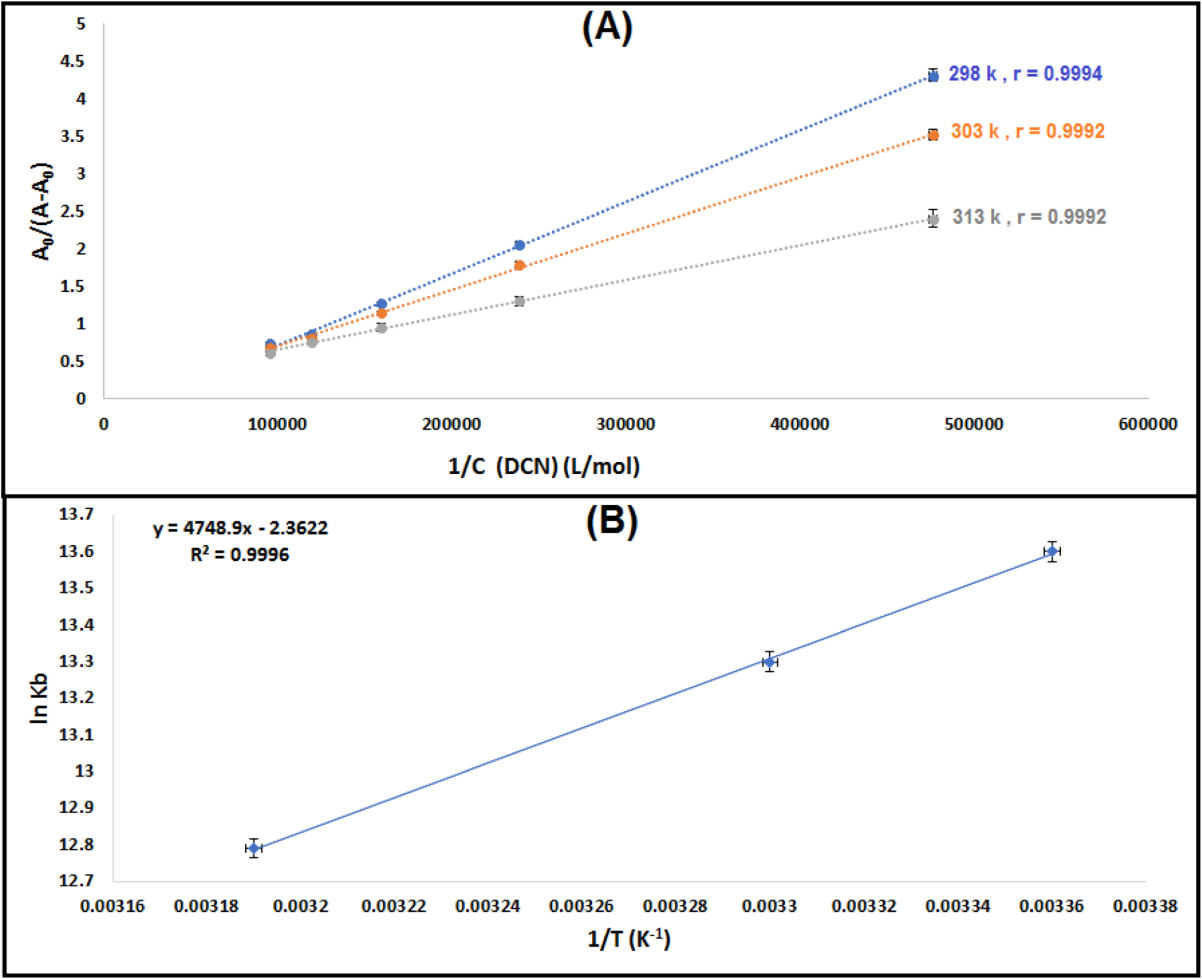
(A) Plot of *A*_0_/(*A* − *A*_0_) *versus* 1/*C*_DCN_ at different temperature settings (*C*_DNA_ = 3.0 × 10^−5^ M), where r is the correlation coefficient. (B) Van't Hoff plot for the ctDNA–DCN complex. Results are reported as mean ± standard deviation (SD) from three separate determinations (*n* = 3).

A plot of 1/*C*_DCN_*vs. A*_0_/(*A − A*_0_) was created at each temperature (298, 303, and 313 K), as shown in Fig. 3A and Table S6. The found linear connection validated the 1 : 1 stoichiometry of the DCN–ctDNA combination. Eqn (1) was used to get the binding constant (*K*_b_) values for the DCN–ctDNA complex, and Table 1 provides a summary of the findings. Throughout the temperature range under investigation, the *K*_b_ values were in the order of 10^5^ M^−1^, indicating a strong binding affinity of DCN for ctDNA. Additionally, these values are similar with those commonly found for groove binders,^16,42,44^ but lower than those of conventional intercalators, including the DNA–EB complex.^45,46^ This lends credence to the idea that DCN and ctDNA interact *via* a groove binding mechanism. Such affinity values, while sufficient for mechanistic characterization, do not alone imply functional relevance in a biological or therapeutic context.

**Table 1:** Assessment of the binding constants (*K*_b_) at three different temperatures and thermodynamic parameters of ctDNA–DCN complex

T (K)	*K*_b_ a (M^−1^) ± SD	Δ*H*^0^ (kJ mol^−1^)	Δ*S*^0^ (J mol^−1^ K^−1^)	Δ*G*^0^^b^ (kJ mol^−1^)	Δ*G*^0^^c^ (kJ mol^−1^)
298	7.7 × 10^5^ ± 0.57	−39.48	−19.64	−33.62	−33.69
303	5.5 × 10^5^ ± 0.72			−33.53	−33.50
313	3.6 × 10^5^ ± 0.41			−33.33	−33.28

a Binding constants are reported as mean ± standard deviation (SD) from three separate determinations (*n* = 3).

b Δ*G*^0^ = *RT* ln *K*_b_.

c Δ*G*^0^ = Δ*H*^0^ − *T*Δ*S*^0^.

Although the present results demonstrate that DCN can associate with ctDNA *via* minor groove binding under *in vitro* conditions, this interaction should not be interpreted as evidence of a biologically relevant genomic target *in vivo*. The binding constants obtained (on the order of 10^5^ M^−1^) are characteristic of non-intercalative groove binders and fall within a range commonly reported for small molecules that exhibit detectable DNA affinity without established cellular or pharmacological consequences. Importantly, DCN is a targeted, irreversible EGFR tyrosine kinase inhibitor, and its anticancer activity is well established to arise predominantly from covalent modification of the EGFR active site. No experimental data in the present study address intracellular drug concentrations, nuclear localization, or DNA engagement in a cellular context. Accordingly, the DNA interaction characterized here is best regarded as a physicochemical property observed under controlled experimental conditions rather than as a demonstrated contributor to therapeutic efficacy or gene regulation.

The binding mode assignment in the present study is based on a combination of spectroscopic signatures, viscosity measurements, competitive displacement assays, and computational analyses, all of which consistently point toward a predominant minor groove association. However, it should be emphasized that these classical experimental techniques provide indirect evidence and are not capable of unambiguously excluding mixed or shallow intercalative binding modes. The observed hyperchromism without a pronounced bathochromic shift, minimal viscosity change, and preferential displacement of a minor groove probe are widely accepted indicators of groove binding, yet they primarily reflect dominant interaction behavior rather than absolute binding exclusivity. Accordingly, while the collective data support minor groove binding as the favored interaction mode for dacomitinib under the conditions studied, the possibility of minor contributions from alternative binding geometries cannot be entirely ruled out.

### 3.3. Assessment of the main interaction forces and thermodynamic parameters

The four primary non-covalent interactions that aid in the binding of biomacromolecules to small molecules are hydrogen bonding, electrostatic forces, hydrophobic interactions, and van der Waals forces.^47^ Furthermore, the values and signs of the entropy (Δ*S*^0^) and enthalpy (Δ*H*^0^) changes frequently provide information about the nature of these binding forces. For example, van der Waals forces and/or hydrogen bonds are probably the main interactions when both Δ*S*^0^ and Δ*H*^0^ are negative. On the other hand, hydrophobic interactions are usually the main forces if both Δ*S*^0^ and Δ*H*^0^ are positive. Conversely, when Δ*S*^0^ is positive and Δ*H*^0^ is near zero, electrostatic interactions are assumed.^48^ The thermodynamic parameters involved in the binding of DCN with ctDNA, such as Gibbs free energy change (Δ*G*^0^), Δ*S*^0^, and Δ*H*^0^, were determined using the Van't Hoff equations (2) and (3):^11^2
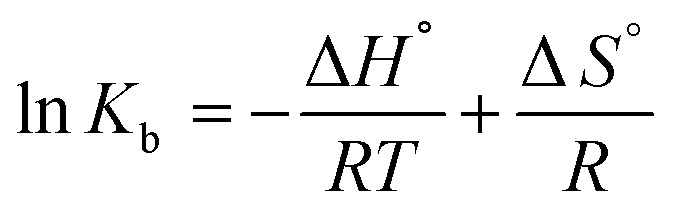

3 Δ*G*° = Δ*H*° − *T*Δ*S*°

where, *R* represents a gas constant.

Table 1 exhibits the Δ*H*^0^ and Δ*S*^0^ values, which were determined using the slope and intercept of the van't Hoff plot of ln *K*_b_ against 1/*T* (Fig. 3B and Table S7). Since Δ*G*^0^ is smaller than zero (negative values), it was concluded that ctDNA and DCN had a spontaneous binding relationship. The negative Δ*S*^0^ and Δ*H*^0^ values are usually due to hydrogen bonding and van der Waals forces. Therefore, hydrogen bonding and van der Waals forces may be the primary binding factors for the DCN–ctDNA relationship, as demonstrated by the molecular docking experiments.

While individual spectroscopic or computational signatures of minor groove binding have been widely reported for classical DNA-binding agents, their convergence in the present system is notable given the pharmacological design of DCN. Unlike traditional groove binders, DCN was not developed to target nucleic acids, yet the combined spectroscopic, hydrodynamic, thermodynamic, docking, and molecular dynamics data consistently indicate a stable minor groove association driven by hydrogen bonding and van der Waals interactions rather than intercalation or nonspecific electrostatics. This observation emphasizes that minor groove recognition can arise from molecular shape complementarity and directional non-covalent interactions even in compounds optimized for protein binding. The present findings therefore extend beyond a compound-specific characterization and illustrate broader principles relevant to drug–DNA interaction studies, particularly in the context of targeted therapeutics where unintended nucleic acid interactions may contribute to secondary biological effects.

### 3.4. *In silico* analysis of DCN

This section will describe the *in silico* analysis of DCN, which is already known for its anti-cancer properties. We have attempted through various computational means, from molecular orbital analysis to ligand–DNA interaction, to portray its electronic property, reactivity, as well as binding sites of anchorage on the DNA of this system. The findings are then rationalized by graphical representations, diagrams and binding affinity data while attempting to unveil the fundamental principles determining the selectivity and stability of its molecular interactions. This multimodal approach expands the possibilities for DCN application as a promising therapeutic agent for DNA-based treatments in the field of molecular oncology.

#### 3.4.1. Electronic properties of DCN: frontier molecular orbital analysis (HOMO–LUMO)

**Fig. 4:**
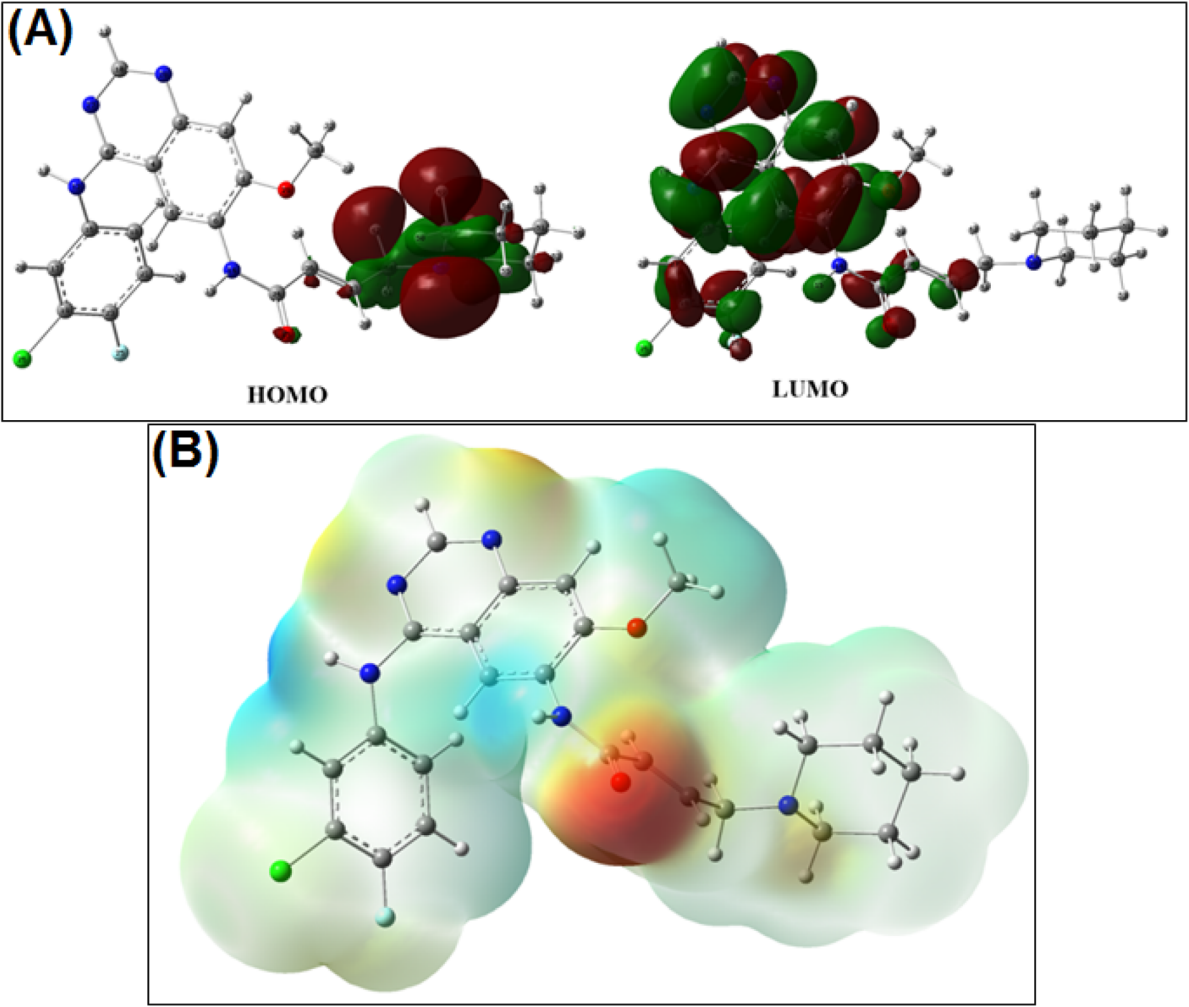
(A) Representation of the HOMO and LUMO orbitals of dacomitinib. (B) Electrostatic potential surface of dacomitinib: Visualization of reactive sites for interaction with DNA.

The frontier molecular orbitals of DCN were calculated to obtain qualitative insight into its electronic structure and potential for non-covalent interactions. As shown in Fig. 4A, the HOMO is primarily localized over the aromatic and heterocyclic regions of the molecule, while the LUMO is distributed across electron-deficient moieties, reflecting an overall delocalized electronic framework. The calculated HOMO–LUMO energy gap indicates moderate electronic stability, which is consistent with the ability of the molecule to participate in weak intermolecular interactions.

It should be emphasized that HOMO–LUMO energies do not predict DNA binding affinity or biological reactivity. Instead, this analysis provides a general electronic context supporting the experimentally observed weak, non-covalent interaction between DCN and DNA, as inferred from spectroscopic and thermodynamic measurements.

#### 3.4.2. Molecular electrostatic potential (MEP) analysis

The molecular electrostatic potential (MEP) surface of DCN was generated to visualize regions of relative electron density that may participate in electrostatic complementarity during non-covalent association. As depicted in Fig. 4B, electron-rich regions are localized around heteroatoms capable of hydrogen bonding, whereas relatively positive regions are associated with hydrogen-deficient aromatic surfaces.

This distribution suggests that dacomitinib possesses spatially separated regions capable of forming weak electrostatic and hydrogen-bonding interactions with the DNA minor groove environment. However, MEP analysis does not imply sequence selectivity, biological targeting, or preferential DNA recognition. Rather, it provides a qualitative framework that complements the experimental evidence supporting minor groove association.

#### 3.4.3. Noncovalent interaction (NCI) analysis

**Fig. 5:**
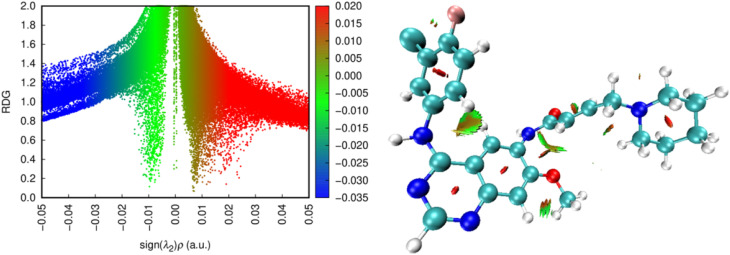
NCI analysis of dacomitinib: non-covalent interactions visualized by the RDG *vs.* sign(*λ*_2_)*ρ* diagram and spatial isosurfaces.

Noncovalent interaction (NCI) analysis was performed to visualize the nature of the intermolecular forces stabilizing the dacomitinib–DNA complex obtained from docking and molecular dynamics simulations. As shown in Fig. 5, the interaction regions are dominated by green isosurfaces, characteristic of weak van der Waals interactions, with limited contributions from hydrogen bonding and negligible indications of strong repulsive or intercalative interactions.

These features are consistent with a non-intercalative binding mode and support the spectroscopic observations of hyperchromism without bathochromic shift and minimal viscosity change. The NCI analysis does not serve as independent proof of binding mode but rather corroborates the experimentally inferred predominance of weak, groove-associated interactions.

Taken together, the HOMO–LUMO, MEP, and NCI analyses provide qualitative electronic and interaction-level context that supports, but does not supersede, the binding mode conclusions derived from experimental spectroscopy, hydrodynamic measurements, and molecular dynamics simulations.

### 3.5. Dacomitinib–DNA interactions analysis: molecular docking, structural study, and interaction diagrams

**Fig. 6:**
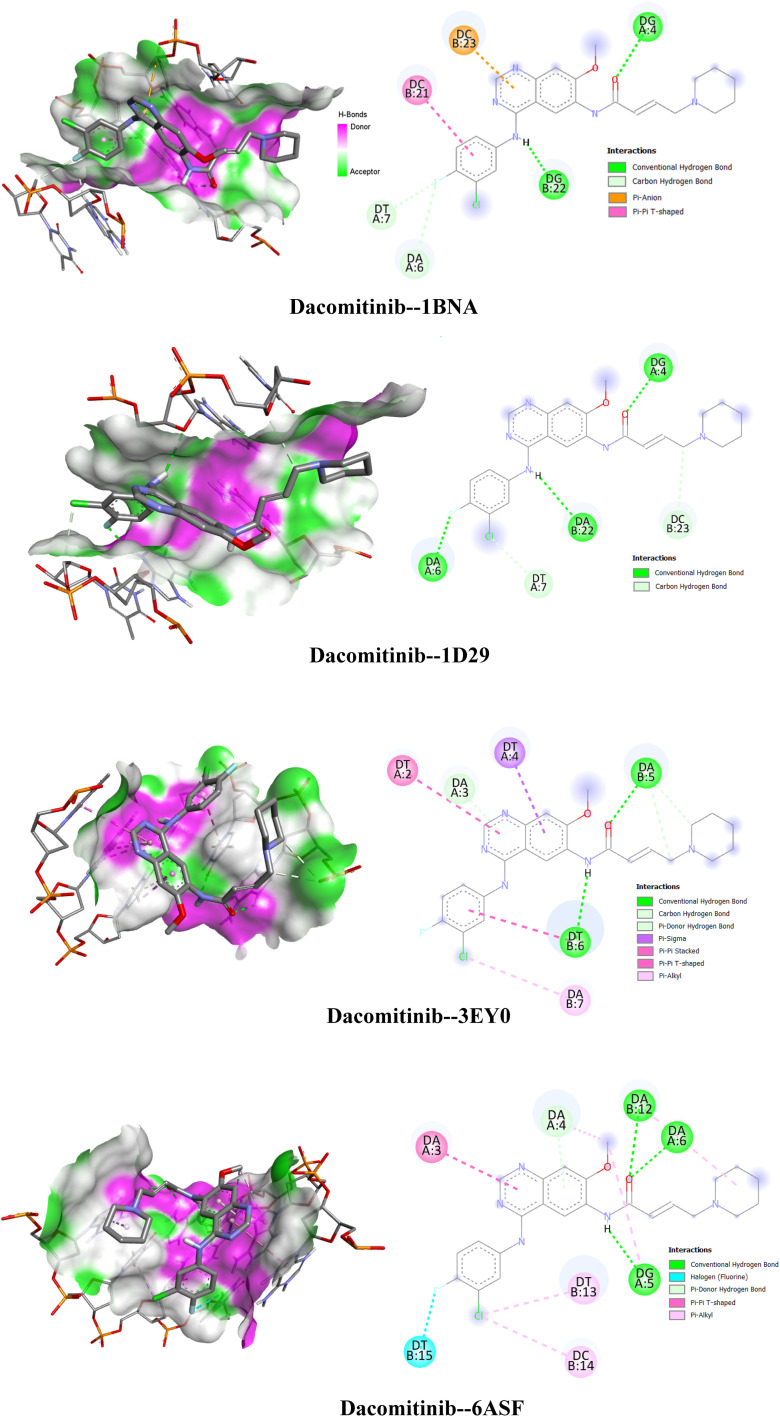
Interactions of dacomitinib with different DNA structures.

In this section, we analyze the interaction between a ligand (Dacomitinib) and different DNA structures represented by the PDB codes: 1BNA, 1D29, 3EY0 and 6ASF. With the help of 3D isometric and interaction schema, the various types of interactions stabilizing them were examined, in particular, hydrogen bonds and π–π interactions are considered. Such analyses expand our knowledge on the specificity of DCN binding to DNA and are beneficial to the evaluation of its ability in biomedical applications like gene therapy and DNA nanotechnology. The results of this study provide valuable data for the design of better responsive ligands that could target particular DNA sites for therapeutic purposes. Table 2 shows DCN's DNA-binding affinity and offers its interactions compared among the various PDB entries; the 3D structures of DCN–DNA complexes with these individual structures are depicted in Fig. 6.

**Table 2:** Affinity of dacomitinib for DNA: comparison of interactions according to PDB codes

DNA model (PDB ID)	Docking affinity a (kJ mol^−1^) ± SD	DNA sequence (5′→3′)	DNA length (bp)	Docking grid parameters (center/size)
1BNA	−33.47 ± 0.47	CGCGAATTCGCG	12	(14.780, 20.976, 8.807)/(60, 60, 110)
1D29	−33.89 ± 0.52	CGTGAATTCACG	12	(14.920, 20.905, 8.820)/(60, 60, 110)
3EY0	−28.87 ± 0.38	ATATATATAT	10	(16.394, 10.415, 90.220)/(80, 60, 60)
6ASF	−32.22 ± 0.75	CCAAGATAG	9	(16.394, 10.415, 90.220)/(80, 60, 60)

a Docking affinities are reported as mean ± standard deviation (SD) from three independent docking runs (*n* = 3) using identical grid parameters for each DNA model.

DNA affinity values (in kcal mol^−1^) of the different DNA–ligand complexes' cues are presented in Table 2 indicated by their corresponding PDB codes and also for the clarity of the readers and for comparison with the experimental measured strength at which the ligand binds DNA. The structure with PDB code 3EY0 is known to possess the lowest affinity among the set (−28.87 kJ mol^−1^) and indicates a weak and unstable binding of the ligand with the DNA. Such effects could be due to low degree of structural complementarity between the ligand and the binding site, or to ligand features that make recognition not of optimal efficiency.

Indeed, PDB entries 1D29 and 1BNA show stronger affinities (−33.89 and −33.47 kJ mol^−1^, respectively), suggesting stronger and stable interactions. These values indicate that the array elements under study have a higher affinity to the DNA, presumably because of improved identification of the binding sites and because of the involvement of more specifically chemical force interaction, such as hydrogen bonding and ionic ones. PDB 6ASF bound ligand possesses an intermediate affinity (−32.22 kJ mol^−1^) indicating a mean interaction which could be useful in the contexts where rapid dissociation of the ligand–DNA complex was required like molecular dynamics studies, competitive binding assay *etc.* In conclusion, ligands such as 1D29 and 1BNA with near −8 kcal mol^−1^ affinities seem to be better suited for use as strong and stable DNA binders, for example, for replication inhibition or structural DNA work. In contrast, the lower-ability 3EY0 ligand may be more suitable for samples in which a less stable interaction is desired, such as competitive binding assays or cases necessitating a fast disassembly of the complex.

In Fig. 6 complexes of DCN (1BNA, 1D29, 3EY0 and 6ASF) with DNA structures with PDB codes are depicted and these are interacted with complexes. In the figures, each panel provides a three-dimensional model of the DCN complexed with DNA and diagrams showing specific interactions of molecules. The ligand of dacomitinib–1BNA was superposed against the DNA and communication between the two ligands was visible. The predominant interactions are classical hydrogen bonds (green) and one π–π stacking interaction (purple). These interactions include hydrogen bonding between hydrogen and oxygen/nitrogen atoms of DCN and the nitrogenous bases of DNA. With dacomitinib–1D29, for which the ligand is also depicted in grey, binding occur in a different configuration than with DB04760, as discussed in the next section. Hydrogen bonds and π–π interactions as well as possibly hydrophobic interaction between the molecules play a role in the stabilization of this complex. In the dacomitinib–3EY0 complex it looks different, probably because the DNA is structurally different. New hydrogen bonds and π–π interactions, and quasivan der Waals contacts or metal ion interactions (though these are not explicitly shown) are found in the interaction diagrams. The π–π stacking interactions are mainly responsible for the ligand–DNA binding stability. Finally, in dacomitinib–6ASF, although the interacting interface is similar to that in the other complexes, the orientation of the ligand is subtly different. Electrostatic or hydrophobic force may further stabilize hydrogen bonds and π–π interaction in this complex. The interactions present in these complexes are dominated by hydrogen bonds (H1, H2 and H3) between hydrogen atoms and O/N atoms, and π–π stacking interactions between the aromatic rings of DCN and the nucleobases of DNA. Although not shown, hydrophobic interactions also contribute to stabilization of the complex in water. Dacomitinib interacts with the various DNA motifs in different ways and configurations, thus reflecting the diversity in DNA structures and how it impacts binding count. That diversity may also influence how stable and functional DCN is, as a ligand. The capacity of DCN for strong binding to DNA renders it particularly attractive for biological or therapeutic applications, such as oncology or genetic studies, in which strong interaction with specific DNA sequences is required. Finally, such figure illustrates the different modes of interaction between DCN and DNA, which is important to understand how this ligand can be employed to target DNA structure and function in other applications.

### 3.6. Molecular dynamics simulations

In order to better verify the docking results and the stability of protein–ligand complexes, all-atom molecular dynamics (MD) simulations were conducted with GROMACS 2021. The systems consisted of the DNA-binding protease (PDB ID: 1D29) in three states: apo (1D29-free), ligand bound (dacomitinib-1D29), and ligand in solvent (dacomitinib-free). The protein topology was constructed with the AMBER99SB-ILDN force field, while the ligands force field parameters were calculated using the General AMBER Force Field (GAFF) with the ACPYPE script. The systems were solvated in water according to the TIP3P model in a cubic simulation box (pbc) with a minimum distance around 10 angstrom between any protein atom and the simulation box. The systems were neutralized by adding the adequate number of Na^+^ or Cl^−^ counterions. Two equilibration stages were run after energy minimization with the steepest descent algorithm: (i) NVT (ensemble with the number of particles, the volume, and the temperature constant) for 100 ps with V-rescale thermostat at 300 K and (ii) NPT (ensemble with the number of particles, the pressure, and the temperature constant) for 100 ps with Parrinello–Rahman barostat at 1 atm. The production MD simulations were carried out for 100 ns using a 2 fs integration time step with bonds restrained using LINCS. Coordinates were saved every 10 ps to analyze trajectory.^32,49,50^

#### 3.6.1. Root mean square deviation (RMSD)

**Fig. 7:**
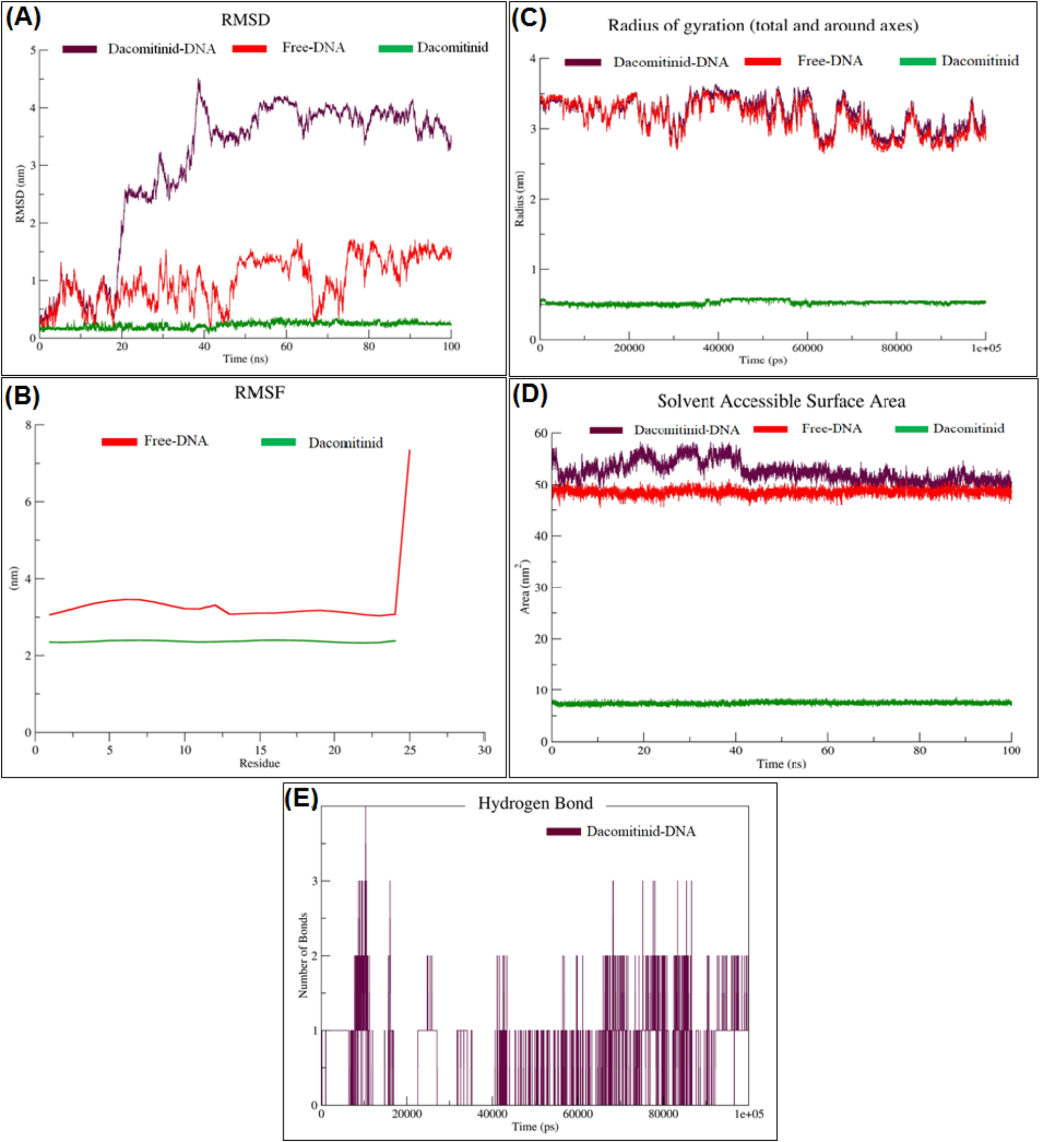
(A) RMSD over 100 ns for the dacomitinib–DNA complex, free DNA, and free dacomitinib. (B) RMSF analysis of free DNA and free dacomitinib at 100 ns molecular dynamics simulation. (C) Temporal behavior of the radius of gyration (*R*_g_) of dacomitinib, free DNA, and dacomitinib–DNA complex. (D) The solvent accessible surface area (SASA) over 100 ns for free dacomitinib, free DNA, and the dacomitinib–DNA complex. (E) Hydrogen bonds between dacomitinib and DNA during the 100 ns of molecular dynamics simulation. Data are derived from single, well-equilibrated production trajectories and are presented as representative examples; observed trends are consistent with previously reported MD behavior for minor groove-bound DNA–ligand systems.

Average Atomic Position Deviation (AAPD), another popular criterion used in MD studies, quantifies the average shift in the atomic positions in a full molecular system throughout time with respect to an average structure. It can reveal structural stability and conformational transitions of biomolecular complexes during the simulation. The frame-specific RMSD X, denoted as RMSDx is given by:
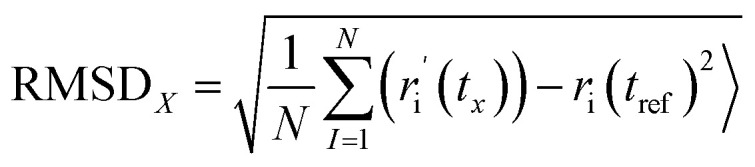


This protocol is superimposing each frame of the trajectory to the reference, which eliminates the translational and roto translational motion; RMSD should therefore contain only the internal structural deviations. RMSD values are calculated for each frame along the trajectory, resulting in a profile that follows the development of structural distortions throughout the simulation. In this work, all RMSD calculations were done at the highest precision respecting the most thorough trajectory processing procedures given in the literature.^51^ The RMSD plots were visualized with XmGrace, which is a popular scientific plotting tool for computational chemistry.^52^ This enabled us to assess and compare the stability of some protein–ligand complexes and find the systems with most stable conformational behavior over the course of simulation. RMSD over 100 ns for the DCN–DNA complex, free DNA, and free DCN are collected in Fig. 7A.

The RMSD plot depicts the structural fluctuation behavior of the three systems, dacomitinib alone (DCN), free DNA, and the dacomitinib–DNA complex, over the 100 ns molecular dynamics simulation. The green trajectory shows that DCN alone in water equilibrates rapidly and remains highly stable around 0.1–0.2 nm (rmsd ≤0.3 nm), reflecting its intrinsic conformational rigidity and structural integrity in the absence of macromolecular interactions.

The red trajectory represents free DNA, which exhibits RMSD values ranging from 0.5 to 1.5 nm, with local peaks and drops, particularly around 60 and 80 ns. These fluctuations reflect moderate structural flexibility, likely due to natural conformational changes such as minor groove breathing, terminal fraying, or loop dynamics in solution.

The purple trajectory corresponds to the dacomitinib–DNA complex and shows a markedly different behavior. Starting from RMSD values similar to free DNA (∼0.5 nm), the complex experiences a rapid increase in deviation between 15 and 35 ns, reaching up to ∼4.0–4.5 nm. This substantial RMSD growth primarily reflects local structural rearrangements of the DNA minor groove to accommodate the ligand, consistent with an induced-fit binding mechanism rather than global destabilization. After ∼40 ns, the RMSD plateaus at ∼4.0 nm, indicating that the complex reaches a new conformational equilibrium. Comparison with free DNA highlights that these deviations result from ligand-induced adjustments rather than intrinsic DNA instability.

Overall, these findings reveal that DCN remains highly stable on its own, free DNA exhibits moderate flexibility, and the DNA–ligand complex undergoes an initial induced-fit transition before settling into a stable bound state. This behavior suggests that dacomitinib extensively engages the DNA scaffold, potentially altering its geometry to achieve favorable and specific binding. Although only single production trajectories were analyzed, the observed trends are consistent with established MD behavior for minor groove-binding ligands, supporting the mechanistic insights described here.

#### 3.6.2. Root mean square fluctuation (RMSF)

Root Mean Square Fluctuation (RMSF) is an important parameter to evaluate the local flexibility of protein residues in MD simulations. Since RMSD describes overall structural squared fluctuations, while RMSF is a per residue mobility, it is particularly useful as a tool for characterization of flexible/immobile regions along the protein. The RMSF of a given residue iii can be calculated using the equation:
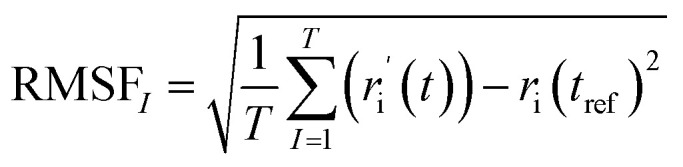


RMSF values were obtained upon best fit of all trajectory frames onto the structure (without ligands) to remove global translational and rotational movements. This process ensures that the resulting fluctuations pertain purely to internal flexibility of the residues, and are not a measure of molecular drift or rotation. The high RMSF areas point to the regions with higher flexibility, generally loops, surface domains and disordered termini. On the other hand, low RMSF corresponds to highly conserved or functional regions, such as α-helices, β-sheets or the active site core. Peaks in the RMSF curve correspond to residues or domains experiencing large fluctuations in thermal motion. In protein systems, as typically found in protein systems, the *N*-terminal and C-terminal tails also tend to present higher fluctuations (being peripheral and typically unstructured).^51^ RMSF analysis of free DNA and free dacomitinib at 100 ns molecular dynamics simulation are presented in Fig. 7B.

The residue-based RMSF analysis gives a clue concerning the flexibility of the molecules during the entire 100 ns simulation. As can be seen in the Fig. 7B, there is a marked discrepancy between the dynamical features of free DNA and the DCN molecule. Free DNA (red) RMSF profile features moderate to high fluctuations along the sequence, with average values near 3.0–3.5 nm, and a noticeable peak of about 7.5 nm at the terminal residue. A sudden increase at the C-terminal end would be expected for an unpaired (or solvent exposed) DNA region, which is known to have higher conformational freedom. These results are consistent with the inherent flexibility of nucleic acid structures in the absence of binding, particularly in their terminal or loop regions.

The RMSF profile for the DCN free molecule (green line) conversely presents a low and flat background through the entire trajectory, with values between 2.2 and 2.4 nm. The lack of pronounced peaks or deviations from the average value indicates that DCN retains a very rigid and stable global state in the aqueous environment with little internal motions. Such rigidity is the hallmark of well folded drug like small molecules with aromatic or heterocyclic cores. Moreover, as shown in the superpositions, free DNA is overall much more flexible in its structure, especially at its ends, while DCN is acting as a conformationally stable ligand. These findings imply that DCN has the possibility to stabilize flexible portions of DNA target when bound to it, which is complementary to restricting their motion upon binding. More comprehensive comparison with the RMSF profile of the DCN–DNA complex would be needed to verify if this stabilization effect is manifested upon binding.

#### 3.6.3. Radius of gyration

Radius of gyration (*R*_g_) is an important MD simulation parameter that describes compactness and atom distribution of a molecular system. For mass-weighted *R*_g_, it is the root mean square applied to distances from the center of mass over the group of interest (for example, a protein or a protein–ligand complex):

The *R*_g_ reflects the compactness state of the molecule, with low *R*_g_ values representing a more compact, folded state and high values a more unfolded, expanded, and/or loss of structural integrity.^53^ It is therefore commonly used to probe the folded state of the protein, the stability of complexes, and the conformational change during the trajectory. Temporal behavior of the radius of gyration (*R*_g_) of dacomitinib, free DNA, and dacomitinib–DNA complex are presented in Fig. 7C.

The *R*_g_ is used to analyze the global compactness and the structural stability of the three molecular systems during a 100 ns molecular dynamics simulation, including free DCN, free DNA, and DCN–DNA complex. The *R*_g_ of free DCN (green curve) is consistently found to be very low, oscillating around ∼0.6 nm over the entire simulation trajectory. This is attributed to the inherent rigidity and close-packed nature of the small molecule, which holds the folded state without much unfolding or restructuring. In stark contrast, free DNA (red curve) displays a moderate fluctuation of its radius of gyration, from about 3.0 to 3.5 nm. Such fluctuations are the hallmark of unbound nucleic acids, caused in large part by flexible loop regions, breathing of base pairs, and transient bending motions.

Interestingly, the *R*_g_ profile of DCN–DNA complex (purple curve) is quite similar to those of the free DNA, which indicates that the binding of DCN does not cause notable global compaction or structural destabilization of DNA. Fluctuation amplitudes and frequencies are similar for both systems, implying that global flexibility and topology of the DNA molecule are not significantly changed by ligand binding. However, some variations on certain time intervals (such as 60–90 ns) seem to suggest that there may be a local stabilizing effect, where DCN may contribute to reduce conformational variability in different parts of the DNA. As conclusion, the gyration radius data shows that DCN is compact and structurally stable as an individual molecule and upon interaction with DNA, which is not being disturbed at the global level of macromolecules. These results are in agreement with the RMSD and RMSF analysis, and indicate that DCN is able to interact with DNA without crashing taking advantage of its flexibility, possibly locally stabilizing without significantly modifying its features.

#### 3.6.4. Solvent accessible surface area (SASA)

The Solvent Accessible Surface Area (SASA) is an important factor for MD simulations, estimating the accessible area of a biomolecule exposed to solvent, usually water. It is a measure of the level of access of atoms or residues of a macromolecule to the surrounding solvent, and therefore is a good indicator of the compactness of the molecule, folding state of a protein, degree of ligand burial, and ruggedness of the conformation during simulation. SASA are usually computed by rolling a virtual probe of solvent, which is a water molecule of ∼1.4 A radius, over the van der Waals surface of the molecule and measuring the traced area. Higher SASA values are related to more solvent exposed residues, possibly indicating an unfolded or flexible region, whereas lower SASA values are associated with more buried or packed structures. It is particularly telling if one's probing extract has caused a ligand-mediated conformation effect or protein folding/unfolding over time.^53^ The SASA over 100 ns was calculated for free dacomitinib, free DNA, and the dacomitinib–DNA complex are shown in Fig. 7D.

The scrutiny of the SASA offers useful information about the degree of molecular exposure to the surrounding aqueous medium and may act as a probe of structural compactness and conformational flexibility during molecular dynamics. SASA was monitored in 100 ns (each) trajectories that belong to the three systems containing: dacomitinib (free), DNA (free), and dacomitinib–DNA complex.

SASA profile of free dacomitinib (green curve) is kept low and steady from 8 to 9 nm^2^ with almost no fluctuation throughout the simulation. This supports that the small molecule is indeed highly packed and folded, hydrophobic in nature, and presents very limited surface area to the solvent.

In comparison, on the free DNA system (red curve) the SASA's are considerably larger yielding an average value of ∼49–50 nm^2^. These values are not unusual for nucleic acids in the unbound, solvated state, despite the fact that a considerable amount of solvent is exposed, with both the phosphate backbone and base pairs. The curve is quite stable, that means there is no significant large conformational collapse or unfolding in the course of simulation.

In dacomitinib–DNA complex (purple curve), SASA is slightly higher than that in the free DNA and oscillates from 51 to 54 nm^2^. This gain in solvent exposure upon ligand binding might suggest local conformational changes in the DNA or of the surface localization of dacomitinib. Importantly, the time behavior of the SASA seems to be unperturbed, indicating that no destabilization or extensive unwinding occurs in the complex. Alternatively, the fact that the values remain largely unchanged suggests that (the complex) retains relatively open (but more stable) structure(s) such that binding may expose new, but again not overall structurally compromising-molecular surface.

Collectively, these findings suggest that despite the fact that dacomitinib has a compact structure by itself, binding of it to DNA increases the total SASA only slightly, suggesting little or no conformational changes or ligand induced exposure factor might be involved. Nevertheless, the SASA curves show that the complex is structurally stable and kinetic consistent during the simulation.

#### 3.6.5. Hydrogen bonds

One of the most important non-covalent interactions, which mediate molecular recognition and contribute to structural stability of protein–ligand complexes, are hydrogen bonds (H-bonds). In MD simulations, analysis of hydrogen bond pathway between a ligand and protein is useful to understand the stability, specificity and strength of binding over time. For example, a stable and repetitive action of hydrogen bonding during the dynamic course is commonly interpreted as response to strong molecular recognition facilitated by structural correspondence of the participants.

Hydrogen bond analysis was conducted throughout the whole 100 ns MD simulation for all protein–ligand complexes in this study. Geometric criteria (3.5 Å for donor–acceptor distances and 120° for donor–hydrogen–acceptor angles) were used to evaluate the plausibility of a hydrogen bond. The hydrogen bond counts were used to track hydrogen bonds dynamically over time. Hydrogen bonds between DCN and DNA during the 100 ns of molecular dynamics simulation are given in Fig. 7E.

The temporal variation of hydrogen bonds between DCN and DNA during the 100 ns MD simulation provides critical information on the stability and flexibility of this complex. On the leg from 0 to 20 ns, the number of hydrogen bonds varies from 1 to 4, suggesting a rather strong and transient contact phase. This indicates that DCN indeed may first actively interact with the DNA, possibly screening for different binding sites and/or orientation, after which it forms many hydrogen bonds. At the intermediate phase (20–60 ns), however, a significant decrease of the hydrogen bond is detected, with repetitive drops to 0. This can indicate a conformational change of the complex, a partial release of the ligand or a modification of binding mode that causes specific hydrogen bond contacts to be transiently lost. In the last part of the simulation (60–100 ns), the interaction is more stable and at least one or two hydrogen bond is stable. This stage presumably corresponds to the transition towards a more stable and energetic favored conformation, at which DCN attains a stronger and more specific interaction with the DNA groove. Although there are not many bonds in total, their long duration in the latter half of the simulation implies a non-random interaction subjected to some kind of targeting, and strengthens the idea of conformationally induced stability. This is in line with known ligand–DNA recognition modes, where not many hydrogen bonds (in contrast to many) appear to suffice in order to provide strength and selectivity. On the whole, taken together, this profile illustrates a dynamic but increasingly stabilizing mode of interaction, validating the notion that DCN is indeed engaged in biologically relevant interactions with the DNA, further justifying the molecular docking and RMSD/*R*_g_.

### 3.7. Integrated analysis of binding mode, methodological considerations and feasibility

To provide a cohesive mechanistic picture, the computational predictions were cross-compared with the experimental observations. Molecular docking identified a preferential binding of the ligand within the DNA minor groove, stabilized predominantly by hydrogen bonds and van der Waals contacts, consistent with the spectroscopic indications of groove selectivity. MD simulations further corroborated this, showing stable complex formation with minimal conformational fluctuations and calculated binding energies that align closely with the experimental *K*_b_ values. Thermodynamic analysis revealed spontaneous binding (negative Δ*G*) and enthalpically favorable interactions, which match the hydrogen bonding and van der Waals contributions observed *in silico*. Collectively, the concordance between docking/MD predictions and experimental spectroscopy and thermodynamics reinforces that the ligand binds selectively to the minor groove, with the computed stabilizing contacts and energetics directly reflecting the measured affinity and free energy changes. This integrated analysis substantiates the mechanistic claim of minor groove binding without invoking alternative binding modes.

The binding mode of DCN toward ctDNA was elucidated through a convergent analysis of spectroscopic, hydrodynamic, thermodynamic, and computational results, allowing alternative interaction mechanisms to be critically assessed. The UV–visible absorption spectra exhibited moderate hyperchromism without appreciable bathochromic shift, a spectral signature inconsistent with classical intercalation, which typically induces hypochromism and pronounced wavelength shifts due to strong π–π stacking between base pairs. This conclusion is further supported by viscosity measurements, which revealed negligible changes in ctDNA relative viscosity upon DCN addition, effectively excluding both classical and partial intercalative binding that would otherwise elongate or distort the DNA helix. Competitive binding assays provided additional discrimination between possible binding modes, as DCN failed to quench EB fluorescence while significantly displacing RB, indicating preferential competition for minor groove sites rather than intercalative positions. Moreover, the weak dependence of DCN–ctDNA interaction on ionic strength argues against nonspecific electrostatic surface adsorption as the dominant binding mechanism. Thermodynamic parameters derived from temperature-dependent binding studies (negative Δ*H*° and Δ*S*° values) further support a binding process governed primarily by hydrogen bonding and van der Waals interactions, consistent with groove association. These experimental findings are in excellent agreement with molecular docking results, which consistently localize DCN within the minor groove without inducing base pair separation, and with molecular dynamics simulations that confirm stable groove accommodation, minimal structural perturbation of the DNA helix, and persistent non-covalent contacts over the simulation timeframe. Collectively, the strong concordance among independent experimental and computational approaches provides compelling evidence that DCN binds predominantly within the minor groove of ctDNA, with negligible contribution from intercalative or nonspecific electrostatic interactions.

The experimental and computational approaches employed in this study differ substantially in terms of time requirements, resource demands, and practical feasibility, and therefore provide complementary rather than interchangeable information. UV–visible absorption, fluorescence spectroscopy, and viscosity measurements are comparatively rapid techniques, typically requiring minutes to hours per experiment, and are well suited for routine screening of DNA–ligand interactions and preliminary binding-mode assessment. These methods rely on standard laboratory instrumentation and modest sample quantities, making them cost-effective and broadly accessible. In contrast, molecular docking and molecular dynamics simulations require greater computational resources and longer execution times, ranging from several hours for docking to multiple days for full molecular dynamics trajectories, depending on system size and simulation length. While computational methods are not constrained by experimental material availability and allow atomic-level visualization of binding modes and interaction stability, their outcomes are inherently model-dependent and sensitive to force-field selection and input structures. Accordingly, spectroscopic techniques provide experimentally grounded evidence of binding behavior, whereas computational approaches offer mechanistic and structural rationalization of these observations. The combined use of both strategies therefore represents a balanced and feasible framework for investigating small-molecule–DNA interactions, with experimental assays enabling rapid assessment and computational analyses providing deeper mechanistic insight.

## 4. Study advantages, limitations, and future perspectives

This study provides a comprehensive investigation of the interaction between DCN and ctDNA, combining multi-spectroscopic, thermodynamic, and computational modeling techniques. A one major strength of this work is its integrated approach, which enables detailed characterization of the binding mode, interaction forces, and dynamic stability of the DCN–DNA complex. The use of molecular docking and dynamics simulations, supported by experimental data, offers valuable structural insights at the atomic level.

While ctDNA serves as a widely accepted and convenient model for probing ligand–DNA interactions, it represents a simplified and relatively uniform B-form duplex, which may not fully capture the structural heterogeneity present in chromosomal, plasmid, or cancer cell DNA. Ct-DNA provides reliable insights into general binding modes, such as minor groove selectivity, and allows quantitative comparison of binding affinities and thermodynamic parameters. However, the extrapolation of these findings to biologically relevant genomic contexts should be made with caution, as sequence composition, local DNA topology, and chromatin packaging can influence ligand accessibility and binding energetics. In the current study, the minor groove binding observed with ctDNA is likely indicative of a general groove preference, but future studies using plasmid DNA, genomic DNA from cancer cells, or alternative model DNAs (*e.g.*, salmon sperm or herring sperm DNA) could provide further validation and explore potential sequence-dependent effects. Overall, while ctDNA results support the mechanistic insights for DCN, complementary investigations in more complex DNA systems will be necessary to fully assess clinical relevance.

It should be noted that the present study does not imply that DNA binding constitutes a primary pharmacological mechanism of DCN, nor does it suggest universal DNA interaction among EGFR inhibitors. Rather, the results demonstrate how integrated experimental–computational analysis can reveal non-obvious DNA interaction propensities in small-molecule drugs, reinforcing the need for mechanistic discrimination between groove binding, intercalation, and electrostatic association when interpreting DNA-binding data.

## 5. Conclusion

This study systematically investigated the binding mechanism of the DCN with ctDNA through an integrated approach employing biophysical techniques, thermodynamic analysis and computational modeling (molecular docking and dynamics simulations). The results demonstrate that DCN preferentially binds to ctDNA *via* a minor groove interaction pattern. Competitive binding assays with established DNA probes yielded distinct responses: RB fluorescence was significantly quenched upon DCN addition, while EB fluorescence remained unaffected. This differential behavior, combined with UV-Vis spectral shifts and increased DNA viscosity, conclusively supports the minor groove binding. A spontaneous binding process (Δ*G* < 0) driven primarily by van der Waals forces and hydrogen bonds was found. The high binding affinity (*K*_b_ = 7.7 × 10^5^ M^−1^ at 298 K) suggests significant pharmacological relevance. The results collectively indicate that DCN preferentially associates with DNA through a minor groove binding mode under *in vitro* conditions, while acknowledging that classical spectroscopic and hydrodynamic assays cannot definitively exclude the presence of mixed or shallow intercalative interactions. This study has revealed several structural and intermolecular aspects of DCN through a combined molecular modeling approach. The quantum study of its electron orbitals, aided with the scanning of its electrostatic potential surface, revealed crucial regions of chemical reactivity that might be involved in biological recognition processes. It was shown by the NCI method that the behavior of DCN depends on a fine-tuned interplay between stabilizing weak interactions and strongly localized repulsions, which would be facilitating the interacting ability of DCN with the biological target like DNA, without leading to any irreversible destruction of the DNA. The quantum chemical analyses presented herein provide qualitative electronic context for the observed non-covalent interactions but are not intended to imply predictive power with respect to DNA targeting or pharmacological activity. *In silico* molecular docking also demonstrates its flexible mode of binding in the grooves of DNA, influencing possible nucleic function. Taken together, these findings indicate that in addition to its enzymatic inhibitors, DCN is a highly promiscuous and medically relevant compound, which could pave the way for novel therapeutic concepts where DNA acts as a second or additional target. In addition MD simulations revealed useful information about the structural dynamic and interaction stability of the DCN–DNA complex. Stable RMSD profile, lower RMSF values, compact radius of gyration and persisted hydrogen bond further confirm that DCN efficiently stabilizes the structure of DNA after binding. The present findings provide mechanistic insight into the non-covalent interaction of dacomitinib with ctDNA, revealing a predominant minor groove binding mode supported by convergent experimental and computational evidence. From a pharmacological perspective, the interaction identified in this study is unlikely to represent a primary mechanism of action of DCN, whose clinical efficacy is well established to arise predominantly from irreversible inhibition of the EGFR tyrosine kinase domain. Rather, the observed association with DNA reflects a weak, non-intercalative minor groove binding interaction under *in vitro* conditions, characteristic of many small molecules that exhibit measurable DNA affinity without demonstrated biological relevance. Minor groove binding mediated by hydrogen bonding and van der Waals interactions, as identified here, is generally less disruptive to DNA structure than intercalation and should not be assumed to exert functional genomic effects in the absence of supporting cellular evidence. Although such interactions may be discussed conceptually as potential off-target associations, the present data do not establish that DCN engages DNA at pharmacologically relevant intracellular concentrations or that such binding contributes to gene regulation, pharmacodynamics, or therapeutic efficacy. Importantly, this study is limited to *in vitro* and *in silico* analyses using calf thymus DNA as a simplified model system, which does not account for sequence specificity, chromatin organization, DNA repair mechanisms, nuclear localization, or cellular pharmacokinetics. Consequently, any extrapolation to *in vivo* genomic interactions, genotoxicity, or clinical outcomes would be premature. Future investigations incorporating cellular models, sequence-selective DNA systems, and direct assessments of genomic integrity would be required to evaluate whether the observed interaction has any functional relevance beyond its physicochemical characterization. Overall, the present work advances molecular-level understanding of how a targeted kinase inhibitor can engage DNA through non-classical minor groove interactions, contributing to fundamental insights into drug–DNA interaction principles while clearly delineating the limitations of biological interpretation.

## Conflicts of interest

The authors declare that they have no known competing financial interests or personal relationships that could have appeared to influence the work reported in this paper.

## Supplementary Material

RA-016-D5RA09242F-s001

## Data Availability

All data supporting this study are provided in the main article and the supplementary information (SI). Supplementary information is available. See DOI: https://doi.org/10.1039/d5ra09242f.
