# Supplementary material for: Multimodal investigation of dacomitinib–calf thymus DNA binding interaction: insights from spectroscopy, thermodynamics, and in-silico studies
Source: RSC Adv. 2026 Jan 26;16(6):5437–54. doi: 10.1039/d5ra09242f (PMC12834094; doi:10.1039/d5ra09242f)
Supplement: RA-016-D5RA09242F-s001 [file RA-016-D5RA09242F-s001.pdf]

Supplementary Information (SI) for RSC Advances.
This journal is © The Royal Society of Chemistry 2026

Supplementary Information

Multimodal Investigation of Dacomitinib-Calf Thymus DNA Binding Interaction: Insights from Spectroscopy, Thermodynamics, and In-Silico Studies

Manal A. Alossaimi [a*], Taibah Aldakhil [a], Heba Elmansi [b], Fathalla Belal[b], Galal Magdy [c,d*]

[a] Pharmaceutical Chemistry Department, College of Pharmacy, Prince Sattam bin Abdulaziz University, Al-Kharj 11942, Saudi Arabia.

[b] Pharmaceutical Analytical Chemistry Department, Faculty of Pharmacy, Mansoura University, Mansoura, 35516, Egypt.

[c] Pharmaceutical Analytical Chemistry Department, Faculty of Pharmacy, Kafrelsheikh University, Kafrelsheikh, 33511, Egypt.

[d] Department of Pharmaceutical Analytical Chemistry, Faculty of Pharmacy, Mansoura National University, Gamasa, 7731168, Egypt.

***Correspondence:** Manal A. Alossaimi: m.alossaimi@psau.edu.sa , Galal Magdy: galal_magdy@pharm.kfs.edu.eg

Table S1: Raw data for UV-Vis spectra of DCN-ctDNA complex using 5 increasing concentration of DCN and a fixed concentration of ctDNA (30.0 μM)

DCN Concentration (μM)	Absorbance [a] (at 260 nm)
0	0.18
2.1	0.23
4.2	0.28
6.3	0.33
8.4	0.38
10.5	0.43

[a] Mean of three different determinations.

Table S2: Raw data for the effect of variable concentrations of DCN (0 – 10.5 μM) on the viscosity of ctDNA (30.0 μM) in Tris-HCl buffer

t_0 [a] (Sec)	t [a] (Sec)	$t-t_0$	$t-t_0/t_0$	η_0 DNA	t [a] Drug (Sec)	t_0 [a] DNA (Sec)	$t-t_0$	$t-t_0/t_0$	η	$(\eta/\eta_0)^{1/3}$	Drug conc. (μM)	DNA conc. (μM)	r	$(\eta/\eta_0)^{1/3}$
20	20.2	0.2	0.01	0.01	26.73	26	0.73	0.0281	0.028	1.2	0	30.0	0	1.2
					26.75		0.75	0.0288	0.029	1.21	2.1		0.07	1.21
					26.8		0.8	0.0308	0.031	1.22	4.2		0.14	1.22
					26.82		0.82	0.0315	0.032	1.21	6.3		0.21	1.21
					26.85		0.85	0.0327	0.033	1.23	8.4		0.28	1.23
					26.89		0.89	0.0342	0.034	1.22	10.5		0.35	1.22

[a] Mean of three different determinations.

Table S3: Raw data for Fluorescence emission spectra of the EB-ctDNA complex in presence and absence of DCN at 298 K. C(ctDNA): 30.0 μM; C(EB): 1.2 × 10^{-3} M; C(DCN): 0, 2.1, 4.2, 6.3, 8.4, 10.5 μM

DCN Concentration (μM)	Fluorescence intensity [a] (λ_{ex}/ λ_{em}= 525/624 nm)
0	430
2.1	428
4.2	425
6.3	425
8.4	424
10.5	424

[a] Mean of three different determinations.

Table S4: Raw data for fluorescence emission spectra of the RB-ctDNA complex in presence and absence of DCN at 298 K. C (ctDNA): 30.0 μM; C(RB): 2.0×10^{-3} M; C(DCN): 0, 2.1, 4.2, 6.3, 8.4, 10.5 μM

DCN Concentration (μM)	Fluorescence intensity [a] (λ_{ex}/ λ_{em}= 465/577 nm)
0	680
2.1	610
4.2	580
6.3	550
8.4	510
10.5	490

[a] Mean of three different determinations.

Table S5: Raw data for the effect of the ionic strength of NaCl on the absorbance of ctDNA-DCN complex. Concentrations of DCN and ctDNA were 6.3×10^{-6} M and 3.0×10^{-5} M, respectively. The concentrations of NaCl: 0, 0.01, 0.02, 0.03, 0.04, 0.05, 0.06, 0.07 M.

NaCl Concentration (M)	Absorbance [a] (at 260 nm)
0	0.323
0.01	0.312
0.02	0.32
0.03	0.316
0.04	0.311
0.05	0.329
0.06	0.319
0.07	0.325

[a] Mean of three different determinations.

Table S6: Raw data the plot of $A_0/(A-A_0)$ versus $1/C_{DCN}$ at different temperature settings (C_{DNA} = 3.0 × 10^{-5} M)

**298 K (25 °C)**					
**A_0 [a]**	**A [a]**	**(A-A_0)**	**A_0/(A-A_0)**	**DCN Conc. (μM)**	**1/ DCN Conc. (L/mol)**
0.201	0.258	0.057	4.3125	2.1	476190.476
	0.314	0.113	2.04950	4.2	238095.238
	0.378	0.177	1.26219	6.3	158730.159
	0.453	0.252	0.8625	8.4	119047.619
	0.493	0.292	0.7289	10.5	95238.0952
**303 K (30 °C)**					
**A_0 [a]**	**A [a]**	**(A-A_0)**	**A_0/(A-A_0)**	**DCN Conc. (μM)**	**1/ DCN Conc. (L/mol)**
0.207	0.255	0.048	3.5263	2.1	476190.476
	0.308	0.101	1.7787	4.2	238095.238
	0.371	0.164	1.1356	6.3	158730.159
	0.447	0.24	0.7976	8.4	119047.619
	0.491	0.284	0.6884	10.5	95238.0952
**313 K (40 °C)**					
**A_0 [a]**	**A [a]**	**(A-A_0)**	**A_0/(A-A_0)**	**DCN Conc. (μM)**	**1/ DCN Conc. (L/mol)**
0.075	0.143	0.068	2.4124	2.1	476190.476
	0.18	0.021	1.3215	4.2	238095.238
	0.215	0.014	0.95763	6.3	158730.159
	0.233	0.032	0.75678	8.4	119047.619
	0.25	0.049	0.6323	10.5	95238.0952

[a] Mean of three different determinations.

Table S7: Raw data for the Van't Hoff plot for the ctDNA-DCN complex

**1/T (K^{-1})**	**K_b (M^{-1})**	**lnK_b**
0.00336	7.7×10^5	13.6
0.0033	5.5×10^5	13.3
0.00319	3.6×10^5	12.79
